# Neurogenesis after Spinal Cord Injury: State of the Art

**DOI:** 10.3390/cells10061499

**Published:** 2021-06-15

**Authors:** Roxana Rodríguez-Barrera, Monserrat Rivas-González, Julián García-Sánchez, Daniel Mojica-Torres, Antonio Ibarra

**Affiliations:** Centro de Investigación en Ciencias de la Salud (CICSA), Facultad de Ciencias de la Salud, Universidad Anáhuac México Norte, Avenida Universidad Anáhuac 46, Lomas Anáhuac, Huixquilucan C.P. 52786, Estado de México, Mexico; roxana.rodriguezb@anahuac.mx (R.R.-B.); monserrat.rivas0202@gmail.com (M.R.-G.); juliangarcia011@gmail.com (J.G.-S.); danielmt94@gmail.com (D.M.-T.)

**Keywords:** neurogenesis, SCI, inflammation

## Abstract

Neurogenesis in the adult state is the process of new neuron formation. This relatively infrequent phenomenon comprises four stages: cell proliferation, cell migration, differentiation, and the integration of these cells into an existing circuit. Recent reports suggest that neurogenesis can be found in different regions of the Central Nervous System (CNS), including the spinal cord (SC). This process can be observed in physiological settings; however, it is more evident in pathological conditions. After spinal cord injury (SCI), the activation of microglial cells and certain cytokines have shown to exert different modulatory effects depending on the presence of inflammation and on the specific region of the injury site. In these conditions, microglial cells and cytokines are considered to play an important role in the regulation of neurogenesis after SCI. The purpose of this article is to present an overview on neural progenitor cells and neurogenic and non-neurogenic zones as well as the cellular and molecular regulation of neurogenesis. Additionally, we will briefly describe the recent advances in the knowledge of neurogenesis after SCI.

## 1. Introduction

Spinal cord injury (SCI) is a damage that causes permanent neurological deterioration of the motor, sensory, and autonomic functions of the central nervous system (CNS) [1]. The incidence of traumatic SCI in North America, Australia, and western Europe is 39, 16, and 15 cases per million individuals, respectively. The prevalence of non-traumatic SCI in Canada and Australia is 1227 cases per million individuals and 364 cases per million individuals, respectively. In North America, between 2010 and 2014, the main causes of SCI included traffic accidents (38%), falls (31%), and sports (10–17%) [2].

The damage inflicted to neural tissue after SCI is induced by primary and secondary mechanisms, inducing self-destructive changes that progressively deteriorate medullary function and may eventually become irreversible. These mechanisms include cell membrane structural loss of neurons and axons, pH alterations, and edema that results in blood flow reduction to the medullary parenchyma. Additionally, the activation of the innate and adaptive response, as well as expression of inflammatory cytokines, play a key role in the pathogenesis and progression of SCI and may determine the clinical outcome [1,2].

Different treatment strategies have been developed, including surgical, pharmacological, and neurophysiological interventions to enhance the functional recovery in affected patients [3]. Although the current treatments for SCI demonstrate certain improvement effects, there is no actual cure for this pathology. For this reason, neurogenesis and pathophysiology in SCI have been receiving increasing attention as they may unveil key factors in developing an effective treatment for this kind of disorder. Neurogenesis induction is the topic of several investigations on neurodegenerative diseases. At the moment, there is interesting information on the factors participating either in enhancing or inhibiting this phenomenon. One of the main factors collaborating in the neurogenic process is the immune system, which, either in physiological or pathophysiological conditions, provides elements that intervene in neurogenic processes in such a way that, if modulated, could improve neurogenesis and thus functional recovery [4]. These beneficial effects have been observed in pathological conditions such as cerebral ischemia or SCI [4,5]. In the latter, the important role of the immune system as an instrument to generate or even boost neurogenesis has been demonstrated. This review is focused on describing some of the immune mechanisms participating in the neurogenic process after SCI. Aside from this, other non-immune elements involved in neurogenesis will be reviewed. In the first part, we will review some basic information related to neural progenitor cells (NPCs), neurogenic zones, and neurogenic processes and their general regulation. Later on, the pathophysiology of SCI, including the immune reaction and its participation on neurogenesis, will be described. Finally, we will review some of the main regulatory mechanisms of neurogenesis, focusing our attention in those present after SCI.

## 2. Neural Progenitor Cells

Even before the discovery of pluripotent reprogramming in cells [6], a stem cell was considered to have two fundamental properties: the ability to self-renew through cell division (asymmetric division) and the ability to generate specialized cell types in multiple cell lineages [7].

However, it is now known that in several adult organs the stem cells are retained in compartments called niches, which regulate their environment and act as a nest or barrier for stem cells and other specific cells, such as vascular cells [8].

The niches demonstrate active proliferation in tissues that require a high renewal rate, for example, blood [9], epithelia [10], and male gonads [11]; in these sites, an efficient balance between cell loss and cell renewal is allowed. In contrast, in tissues in which cell loss is limited, such as in the liver, teeth, and brain, stem cells are present in a reduced and resting state and can be activated by physiological or pathological means, which protects, nourishes, and regulates the destination of the stem cells [12]. This is regulated by the existence of highly organized structures as well as cellular and molecular signals suitable for the strict control of stem cells, including self-renewal, differentiation, and quiescence. Typically, these niches maintain high protein levels associated with the canonical proliferation signaling pathways, particularly bone morphogenetic protein (BMP), WNT proteins (Wingless contraction “wingless”), and the Notch pathway [13,14]. These signaling pathways accurately regulate the proliferation, quiescence, differentiation, and self-renewal of stem cells. In addition, its particular architecture favors the interaction between stimulation [15].

NPCs are multipotential, meaning they possess the capacity to form specific cellular types of CNS: astrocytes, oligodendrocytes, and neurons [16,17]. NPCs are present in the adult CNS, playing an important role in its maintenance and self-renewal. Interestingly, NPCs have been recognized as one of the responsible cells involved in the generation of brain tumors, furtherly supporting the idea that the balance between survival, growth, and differentiation is a critical aspect of CNS biology [18].

## 3. Neurogenic and Non-Neurogenic Zones

Adult neurogenesis is defined as the process by which new neurons are formed. This includes precursor cell division signaling, their differentiation, settlement, and integration to the functional circuitry [18]. Neurogenic regions can be described as specific brain locations where the production of new neurons is held, while a neurogenic niche is the specific region where the NPCs reside [19]. Their function is related to their permissive cellular microenvironment, which orchestrates the proliferation and differentiation process of neural precursors into a neuronal lineage. Moreover, along with external stimuli, they possess the ability to regulate the migration and maturation of newborn neurons into other CNS regions [20].

In the adult brain, there are only two well-defined neurogenic areas: the subventricular zone (SVZ) and the subgranular area (SGZ) of the hippocampal dentate gyrus (GDH). It has been reported that these areas generate newborn neurons that eventually differentiate into mature granular neurons [21]. NPCs in the lateral ventricle and hippocampal dentate gyrus show astrocytic and neuroepithelial features. These cells generate transit-amplifying progenitors that are also able to induce neurogenesis. The SVZ progeny will migrate to the olfactory bulb, while dentate gyrus neurons will stay in the hippocampus [22].

The regions outside these two sites have been called “Non-neurogenic” zones. Although it is conjectured that neurogenesis takes place only in these two areas, recent reports suggest that neurogenesis in the adult state can be found in other regions of the CNS, such as the amygdala and neocortex [19], cerebellum, striatum, and substantia nigra [23]. Additionally, it has been reported that neurogenesis is also amplified after mechanical damage [24].

## 4. Neurogenesis Regulation

Neurogenesis is regulated through a complex mechanism involving the immune system, neurotransmitters, transcriptional factors, growth factors, and even other processes such as gliogenesis. This topic represents a very extensive research field and therefore we will be focusing primarily on a few immune-related factors and, later, on other elements that have demonstrated consistently to play key roles in neurogenesis regulation.

In the same way that the CNS can influence immunity, the immune system also plays a crucial role in brain development, neuronal differentiation, and synaptic plasticity in physiological conditions [25].

Immune cells can also amplify and suppress neurogenesis. Cytokines such as IL-1β, IL-2, and IL-6 play an important role regulating neuronal functions. The presence of an uncontrolled chronic inflammation during neurogenesis still causes controversy between pro- and anti-neurogenic properties of immune response, which may depend on the duration of the inflammatory response and the milieu by which the microglia, macrophages, and astrocytes are activated.

Certain studies have demonstrated that microglia can determine the outcome of NPC differentiation. In this way, whether the effect of activated microglia on the injured CNS will be favorable or prejudicial is determined by the type of activation [26]. It has been suggested that the proinflammatory activated microglia inhibits neurogenesis; however, the microglia activated by IL-4, or by low levels of IFN-γ, is associated with a Th cell response that induces neurogenesis and axon elongation [27]. Furthermore, IL-4 inhibits nitric oxide production and proinflammatory cytokines secretion such as TNFα and IFNy. Additionally, previous data suggest that IL-4 provides beneficial effects on neural restoration; it has been pointed out that IL-4 increases oligodendrocyte ramification and maturation through microglial interaction [26]. Moreover, it is confirmed that IL-4 can induce axonal outgrowth in ex vivo models. In addition, neurons in the presence of IL-4 promote axonal elongation and restoration of damaged neurons by the activation of neuronal IL-4 receptors that amplify neurotrophic signaling via AKT and MAPK pathways [28]. Additionally, it was demonstrated that IL-4 boosts IGF-1 expression, a particularly important molecule that contributes to neurite extension [26]. In general, IL-4 represents a key factor for tissue maintenance, cell viability, and axonal growth [29].

Similarly, it has been demonstrated that IL-4 induces NPC differentiation into doublecortin-expressing neurons (DCX) and increases the expression of BDNF on the choroid plexus, a key molecule in mediating CNS plasticity, specifically neurogenesis [26,30]. IL-4 also reduces the production of nitric oxide (NO) and TNFα, two pro-inflammatory molecules that are strongly associated with neurogenesis suppression [31]. Additionally, IL-4 has been shown to protect hippocampal neurogenesis after immunization with neural-derived peptides (INDP) in experimental autoimmune encephalitis [32]. Overall, these findings suggest that IL-4 plays an important role in increased cell survival and neurogenesis [26].

The production of the anti-inflammatory cytokine interleukin IL-10 is one of the most important immune mechanisms to counteract the damage driven by excessive inflammation [33]. Nevertheless, the clear effect of IL-10 on neurogenesis remains a challenging field due to the controversial results observed in some of the recent investigations.

Most of the studies report that the presence of IL-10 delays cell cycle exits and therefore promotes the maintenance of neural progenitors in an undifferentiated state in the normal brain [34,35,36]. Nonetheless, other studies have demonstrated that IL-10 plays a key role in the regulation of adult neurogenesis through a mechanism of action independent of its well-known anti-inflammatory properties. IL-10 regulates the expression of undifferentiated neural progenitor markers (Nestin+, Sox1, Sox2, Mash1), cell cycle activity, and the production of new neuroblasts in the SVZ by activating the phosphorylation of ERK and STAT3 in Nestin+ progenitors [34]. Consistently, when IL-10 levels are reduced, neuronal gene expression becomes more prevalent, causing an increase in neurogenesis [35]. On the other hand, IL-10 has shown different effects in response to cerebral aggression or neurodegeneration. After stroke, a significant correlation was found between IL-10 levels and neurogenesis, suggesting that this cytokine may play an important role in neurogenic processes [5]. Similarly, another study confirmed that, under CNS aggression, the administration of activated T-regulatory cells promoted NPC proliferation via IL-10. These data suggest that IL-10 may play a critical role in neurogenesis in the SVZ after focal ischemia. This same study also demonstrated that the overexpression of IL-10 in the hippocampus increased the number of DCX+ and BrdU+/NeuN+ neurons in the SGZ of mice [36]. Together, these results demonstrate a novel physiologic function of IL-10 in neurogenesis regulation.

In addition to the immune system, other factors regulate neurogenesis. For instance, neurotransmitters such as glutamate, gamma-aminbutyric acid (GABA), acetylcholine (Ach), dopamine, and serotonin (5-HT) are implicated in the development of the adult brain. Neurotransmitters influence cell proliferation and differentiation within neurogenic zones [37,38].

The WNT factor, secreted by astrocytes of the adult neurogenic zones, promotes the proliferation of neuroblasts and regulates neuronal specificity. It has been demonstrated that the inhibition of WNT reduces neurogenesis significantly; however, the overexpression of WNT-3 is able to increase neurogenesis [39]. On the other hand, activation of JAK/STAT and MAPK (protein kinase activated by Mitogens) signaling pathways induce the proliferation of NPCs in the SC [40].

Transcriptional factors play an essential role in the expression of regulatory proteins that promote adult neurogenesis. One of the most characterized factors is SRY-related high mobility group box 2 (Sox2). Sox2 is expressed in both radial and horizontal NPCs and plays a key role in NPC self-renewal. In addition, Sox2 interaction with RMST, a long non-coding RNA, was found to be essential in the lineage outcome of NPCs [38].

Another factor known is forkhead box protein O3 (FOXO3), which represents a direct target of the phosphatidylinositol 3-kinase AKT pathway (PI3K)/protein B, which regulates cell survival functions and cell cycle progression. Recent reports have shown that FOXO3 could inhibit the progression of the cell cycle in the G1/S transition by inhibiting cyclin-dependent kinase transcription factors on the control of p27 transcription, which is also a key regulator in the neurogenesis of mammals [41]. In addition, the absence of the FOXO3 in the SVZ and SGZ leads to failure in the NPCs’ ability to return to the quiescent state, which subsequently causes depletion of the NPC pool [38].

Among neighboring cells, several studies indicate that endothelial and ependymal cells may also play a key role in neurogenesis. It has been demonstrated that high amounts of vascular endothelial growth factor (VEGF) induce both neurogenesis and angiogenesis in the hippocampus, while the blocking of the VEGF signaling pathways increases neurogenesis [42]. When the pigment epithelium-derived factor (PEDF) is released by ependymal and endothelial cells, neurogenesis is stimulated through the activation of Hes1 and HES5 proteins, which are the main mediators of the Notch pathway. In addition, the direct contact of the NPCs with endothelial and ependymal cells, through laminin-integrin interactions [27] and secreted factors such as VEGF or PEDF, is necessary to support and mediate neurogenesis after SCI [43]. Overall, these data indicate that both endothelial and ependymal cells are involved in the regulation of adult neurogenesis.

On the other hand, there are also some mechanisms that negatively control neurogenesis; for instance, it has been reported that the formation of new astrocytes (gliogenesis) occurs in neurogenic and non-neurogenic areas of the CNS, especially after injury [43]. Clear evidence of this process is observed in the formation of an acute astrocytic response, limiting and restricting the inflammatory extent after an injury but also reducing axonal regeneration. In addition, this glial scar formation, conducted by astrocytic cells and collagen, releases proteoglycans and neurofilaments such as vimentin and nestin+ that act as inhibitory molecules of neural growth [44,45]. The glial scar reduces the possibility of grafted cells migrating and integrating into existing circuits [46]. For this reason, most transplant studies are performed during the acute phases 1–2 weeks after the injury [47], and it is more difficult to foresee a therapy based on stem cells in the acute phase than in the chronic one [48].

## 5. Neural Progenitor Cells in the Spinal Cord

Several studies have shown that NPCs in the ependymal channel of turtles, fish (Apteronotus albifrons), and amphibians [49,50] express neural molecular markers in early stages of differentiation; one of the most studied is HuC/D [51]. The presence of this type of cell in non-hominid species has theorized the hypothesis that this type of cell may also be present in mammals. Marichal et al. performed immunohistochemistry studies with newborn mice where the presence of cells expressing HuC/D was observed; however, when determining the expression of NeuN+ (a mature neuron marker) it was found that the ependymal cells that expressed HuC/D did not express NeuN+. In this same study, the expression of molecules that are present in neuronal precursors of neurogenic niches in the brain were analyzed through double labeling [51]. DCX and the polysialylated form of Neural Cell Adhesion Molecule (PSA-NCAM) proteins were expressed in HuC/D positive cells. Despite these results, it is not certain whether all the cells present in the ependymal canal are precursors of neurons, because DCX (marker of early-forming neurons) and PSA-NCAM are also expressed simultaneously in glial cell progenitors. Despite the uncertainty about this type of cell, it has been demonstrated that, when ependymal cells are isolated, they possess the ability to form neurospheres in culture media, to which epidermal growth factor (EGF) and EGF-2 are added [52]. Little is known about the ependymal cell niche in the SC, but the ciliated ependymal cells have been identified in the lumen-contacts in their different classes: cubic, tanycytic, and radial [53].

To know the identity of NPCs that show neuronal properties in the adult SC, fluorescent markers such as BrdU+, as well as the marking of fluorescent cells in transgenic mice that express reporter genes such as LacZ or GFP (fluorescent green protein), have been used. These markers suggest that most cells initiate as neurospheres and reside near the central channel, representing a close relationship with the ventricular system [30]. In contrast, other studies have proposed that a number of clones can be propagated from the middle and lateral zone of the SC [54]. The existence of two different cell populations has been suggested: a multipotent population with the capacity of extended self-renewal residing near the canal and another population with a more limited self-renewal capacity restricted to the lineages of the glia present in the parenchyma [55].

Finally, published studies by Habib et al. in 2016 showed unusual populations of GABAergic newborn neurons that express Gad1 and Gad2 markers by seq-div technique in the SC. Additionally, gene expression was compared between the rostral migratory stream of the olfactory bulb and the SC; interestingly 347 neurogenesis related genes were identified including the Pre-B-cell leukemia transcription factor 3 (Pbx3) and Meis Homeobox 2 (Meis2). Nevertheless, more studies are needed to fully understand their specific function in the SC [56].

After SCI, the microenvironment changes and the conditions for neurogenesis are also different.

## 6. Pathophysiology after Spinal Cord Injury

After SCI, a series of anatomical and physiological self-destructive mechanisms are triggered, originating the discontinuity of the medullar parenchyma with long-term sequelae [57].

For its study, SCI has been divided into three phases [58]. The first phase includes immediate neuronal damage due to the hemorrhage and decreased blood flow caused by the initial impact, resulting in ischemia and necrosis [59,60]. During this phase, the generated edema in conjunction with the accumulation of intracellular calcium [61], increased glucose concentrations, and a decrease in ATP synthesis, generating an interruption in the electrical flux resulting in spinal shock. The second phase is characterized by the emergence of biochemical alterations such as lipid peroxidation and the accumulation of excitatory amino acids in the injury zone and penumbra area, causing overexcitation and the collapse of neurons [59,62].

Finally, during the third or chronic phase, disturbances in fiber organization such as demyelination, Wallerian degeneration, oligodendrocyte apoptosis, and glial scar formation [63,64].

Temporarily speaking, traumatic SCI can also be divided into acute (<48 h), subacute (48 h to 14 days), intermediate (14 days to 6 months), and chronic (>6 months) phases [65]. One of the main pathophysiological factors participating in tissue destruction, after SCI, is the activation of the immune system, an event participating across all the stages of SCI [66]. During the first hours to days after the initial trauma, a cellular reaction mediated by macrophages, neutrophils, T cells, and reactive astroglia is generated, promoting an exacerbated inflammatory and autoreactive process that causes significant damage to the neural tissue [67,68]. During the inflammatory response, the infiltration of immunological cells is the main contributor of neuronal degeneration and the consequent motor and somatic deterioration [69]. The pro-inflammatory immune response observed after SCI is one of the main factors that could be negatively regulating neurogenesis.

## 7. Immune Reaction after SCI and Its Effect on Neurogenesis

Inflammation can be defined as a cellular and molecular response to stress, infections, or injuries [70]. After activation, the inflammatory process is initiated by resident microglia and astrocytes, as well as by the infiltration of T cells and peripheral macrophages. This reaction triggers a series of inflammatory stimuli in several cell types [71], resulting in the production of different pro-inflammatory cytokines, interferon gamma (IFNγ), tumor necrosis factor alpha (TNFα), interleukin-1 beta (IL-1β) interleukin-18 (IL-18), interleukin-6 (IL-6), neurotransmitters, and reactive oxygen and nitrogen species (peroxide and nitric oxide), as well as chemokines. Normally, microglia in the healthy CNS resides in a resting inactive state. However, in response to injury and infections, the microglia switches to an activated state (M1 phenotype), leading to the release of proinflammatory cytokines such as TNFα, IL-6, and IL-1β [69]. In SCI models, the vascular permeability disruption—a consequence of inflammatory reaction—facilitates the entrance of peripheral immune cells that secrete proinflammatory cytokines such as Il1β and TNFα [72]. These cytokines could negatively regulate neurogenesis by reducing NPC proliferation and neuronal differentiation. In addition, impaired microglia may underpin dysregulated microglial activation. This is a deleterious effect frequently observed in neurological diseases and discloses novel therapeutic targets to promote white matter regeneration [73].

In contrast to its inflammatory-deleterious effects, microglia in an alternative-activated state (M2 phenotype) can be capable of releasing anti-inflammatory cytokines such as IL-4, IL-10, transforming growth factor-β (TGF-β), insulin-like growth factor-1 (IGF-1), and brain-derived neurotrophic factor (BDNF). Several studies have already reported that these molecules mediate neuronal differentiation, migration, and neurogenesis [74]. Therefore, it could be assumed that the immune response could exert a different effect on neurogenesis depending on the activation state of microglia. Namely, while a pro-inflammatory phenotype inhibits neurogenesis, an anti-inflammatory phenotype may support and promote neurogenesis.

After injury, there is a significant increase of NPCs; however, only a few cells survive to reach a functional state, and many of them usually acquire a glial phenotype instead of developing into newborn neurons. Recent studies have suggested that the suppressors of cytokine signaling (SOCS) family members can regulate the immune response in different CNS injury models [75]. SOCS2 has been described as an important factor in newborn neuron survival regulation with potential anti-inflammatory functions in the CNS [76].

It has been reported that overexpression of SOCS2 increases the number of newborn adult hippocampal neurons. Moreover, it has been demonstrated that SOCS2 promotes neurite outgrowth in vitro and in the nerve growth factor (NGF) [77] and epidermal growth factor (EGF) [78] signaling pathways. In addition, Basrai et al. examined neurogenesis as well as the morphology of newborn neurons in endogenous adult hippocampal regions in SOCS2KO mice, reporting there were no differences in the differentiation or proliferation of NPCs, but SOCS2 deficit caused a reduced number of hippocampal mature neurons. In the same study, the authors found an increase in the number of mature spines. These contrasting results indicate that the participation of SOCS2 in neural restoration is quite complex and should be further studied. On the other hand, SOCS3 has been associated with nerve growth limitations after a complete SCI. Genetic modification of SOCS3 have been performed in several studies in an attempt to modify its negative outcome in neurogenesis regulation [76]. A study conducted by Park in 2015, which evaluated the endogenous expression of SOCS3 and their role in neurite outgrowth regulation, demonstrated that the lack of SOCS3 increases dendritic regeneration and prevents demyelination after SCI [79].

## 8. Neurogenesis after SCI

SC restoration after injury represents a very extensive field; therefore, this next section is strongly focused on some of the restorative strategies that have demonstrated promising results after SCI. We focus our review on the most important cells, molecules, and transcription factors involved in improving neurogenesis.

NPCs have been identified especially in brain niches; however, few studies have also addressed this issue in the SC [8]. After SCI, almost two million new cells are produced at the site of injury, having a peak at 3–7 days post injury [80]. In the acute phase of SCI, the proliferation, migration, and differentiation of NPCs to NeuN+ mature neurons are frequent induced processes [70]. Nonetheless, there is scarce information about the origin of these cells as well as on the existence of neurogenic niches in the SC. Regarding the latter, current investigations have placed particular emphasis on the ependymal channel as a possible origin of NPCs. Some studies have reported the proliferation, migration, and differentiation of ependymal cells into NPCs after SCI [81,82].

Ependymal cells are rarely divided in the normal SC; however, it has been demonstrated that after injury a massive increase in their proliferation within the first 24 h is induced [83,84,85]. A large part of proliferating ependymal cells have a parallel division plane to the surface after SCI, suggesting that a newborn cell may remain in the ependymal layer and then migrate [46]. Most of these cells lose their phenotype and begin to express diverse markers, generating different types of cell linages. 

Other cells with neurogenic potential—in the ependymal channel—are the ependymal tanycytes, which should also be studied as a possible origin of NPCs [86,87].

Although the ependymal channel emerges as the main source of neurogenesis, neuroblasts could also derive from meningeal cells [88] or even from other places such as the hippocampus [89]. This topic requires further investigation.

On the other hand, several molecules and transcription factors have been strongly associated with the neurogenic process observed after SCI. A study observed that in normal SC, CXCL12/SDF-1 (stromal cell-derived factor-1) is expressed by the dorsal corticospinal tract and by the meninges, whereas ependymal cells express CXCR4. However, after SCI, infiltrating macrophages and perhaps ependymal cells, both of which express CXCR4, appear to migrate towards both the dorsal corticospinal tract and meningeal sources of SDF1. In addition, another population of CXCR4+ cells remains in the ependymal layer after SCI [90,91]. Moreover, in a study reported by Muller et al., it was found that CXCL12 is able to promote neurite growth in myelin sensitive neurons. Expression and distribution of CXCR4 and CXCR7/RDC1 receptors in dorsal ganglion neurons were assayed in vitro, and it was demonstrated that CXCR4 receptors are present in P6 dorsal root ganglion neurons, mostly in the growth cone and ramification points during the first culture stages, suggesting that the CXCR4 receptor promotes growth and axonal arborization. Interestingly, these in vitro findings were confirmed with a subsequent in vivo experiment where SDF-1 inoculation by intrathecal injection after a hemisection injury evoked corticospinal tract axonal regrowth [92].

Likewise, it has been observed that Sox11, a transcription factor involved in neural development and organogenesis in fetal life, as well as in the differentiation of NPCs during neural development, has an important role in neurogenesis and locomotor recovery after SCI. Guo et al. demonstrated that the introduction of a lentiviral vector containing the Sox11 gene into the injured SC of mice improved locomotor recovery, accompanied by an up-regulation of Nestin/Dcx expression [91]. They also found that Sox11 promoted BDNF expression, which supports NPCs differentiation into neurons [93,94,95].

It has also been suggested that the proliferating ependymal cells that are divided in response to the SCI segregate Notch1, a well-characterized receptor that has been associated with the proliferation responses of NPCs. The functions of Notch signaling in the NPCs have been studied mainly in neurons, oligodendrocytes, and astrocytes during the embryonic development; however, nowadays there is increasing evidence that Notch’s signaling plays a fundamental role in the maintenance and differentiation of the adult CNS [96].

There are also accessory cells that play an important role in neurogenesis after SCI. Microglia/macrophages were reported to support the growth and survival of neurons [95,96]. In particular, it has been observed that these cells are capable of polarizing toward an M2 phenotype and decreasing inflammation. This is an important effect that promotes restorative processes, as well as neurogenesis, axonal remodeling, angiogenesis, oligodendrogenesis, and remyelination [4,97]. In fact, it has been demonstrated that M2 microglia/macrophage phenotype can promote the regeneration of sensory axons in the SCI [98]. In addition, M2 microglia/macrophages that secrete molecules such as IL-10 and GAP-43 have been demonstrated to promote axonal regrowth and motor recovery after SCI [99].

In recent times, the presence of immune cells in the brain were considered as malignant, and in order to avoid deleterious damage they were eliminated or inhibited. Nevertheless, nowadays, many studies have proved that the immune system and CNS are in constant interaction under physiological conditions to maintain homeostasis and provide beneficial effects [100]. Recently it has been demonstrated that protection and restoring mechanisms can be evoked after CD4+ T cells prime CNS autoantigens during the immune response; a phenomenon that was first proposed and named by Michael Schwartz in the late nineteenth century as protective autoimmunity, and is now considered as a physiological response to a CNS damage. Protective autoimmunity depends on a particular CD4+ T cell response to specific neural autoantigens that, under certain conditions, protects, repairs, and restores nerve tissue instead of destroying it [101]. Under this context, many experiments were carried out to demonstrate that activated T cells against CNS compounds are needed to orchestrate neuroprotection after traumatic injury [102]. Diverse research studies have also shown that immune response modulation through protective autoimmunity can improve functional recovery and increase neurogenesis in stroke [5] and SCI models [4,24].

Most of the studies on neurogenesis after SCI have been performed during the acute phase of the injury; however, in chronic stages, evidence of this restorative phenomenon is also present. Previous studies have demonstrated that neurogenesis after chronic SCI is a physiological phenomenon and that it can be boosted by immunizing with neural-derived peptides (INDP). This neurogenic effect is attributed to an increase in anti-inflammatory and regeneration-associated proteins alongside the reduction of pro-inflammatory cytokines, which promote the induction of a favorable microenvironment for neurogenic processes [4,24]. Moreover, INDP promotes restorative effects by inducing the expression of BDNF and GAP-43, two molecules strongly associated with neurogenesis [24,99]. Several studies have previously shown that the BDNF/TrkB pathway plays an important role in the induction of neurogenesis [103]. Similarly, the upregulation of GAP-43 is involved in cell division orientation and it is required to establish neuron maturity [104]. Additionally, these studies demonstrated that INDP increases the expression of IL-4 and IL-10, which have been strongly associated with the induction of neurogenesis as well as with neuroprotective and regenerative actions [4,77].

In vivo reprogramming has acquired increasing attention in the last few years to induce neurogenesis, as it represents a big challenge for the regenerative medicine. A recent study demonstrated the development of newborn neurons from glial cells actively forming new neural circuitry and improving functional activity [105]. In another study, reprogrammed astrocytes with ectopic expression of SOX2 results in an induction of neuroblast DCX+ after SCI [106]. Additionally, these neuroblasts demonstrated the ability to expand and convert into mature neurons capable of forming connections with motor neurons through the p53-p21 signaling pathway [107]. Moreover, it has been reported that most of the induced SOX2 neurons are excitatory motor neurons VGLUT2+, which play an essential role in obtaining better functional results after SCI [108].

Overall, these studies suggest that neurogenesis could be a physiological event in the acute and chronic phases of injury [24]. Nevertheless, the exact origin of these cells remains unknown. On the other hand, these studies emphasize that the ependymal channel is emerging as a possible neurogenic niche due to its potential to induce proliferation, migration, and differentiation of ependymal cells into NPCs after the injury [5,35]. Finally, neurogenesis could be boosted even in chronic stages of injury by INDP; this strategy has been shown to promote a better motor recovery after SCI.

## 9. Conclusions

The complex pathophysiology observed in SCI explains the amount of different therapeutic strategies that aim to modulate the formation of new neurons. Although some studies have shown significant therapeutic potential, there are still enormous knowledge gaps that need further investigation in order to develop a potential cure for SCI. In the way to induce a functional neurogenesis, certain cytokines seem to have promising neurogenic effects [21,24,28,29,30,31]. In the same way, ependymal cells and some molecules and transcription factors appear to be the possible therapeutic targets. Neurogenic niches biology in health or disease states is still a topic that should be further studied in the SC. Finally, studies will be crucial to identify the best way to enhance therapeutic approaches such as INDP in an SCI context involving the induction of its complex inflammatory process in order to improve neuronal survival, CNS plasticity, and neurogenesis.

## Data Availability

Data sharing not applicable.
